# Malignant peritoneal mesothelioma with lymph node metastasis that originated in the transverse colon

**DOI:** 10.1186/1477-7819-12-112

**Published:** 2014-04-23

**Authors:** Yusuke Takehara, Shungo Endo, Yuichi Mori, Kenta Nakahara, Daisuke Takayanagi, Shoji Shimada, Tomokatsu Omoto, Chiyo Maeda, Shumpei Mukai, Eiji Hidaka, Fumio Ishida, Jun-ichi Tanaka, Shin-ei Kudo

**Affiliations:** 1 Digestive Disease Center, Showa University Northern Yokohama Hospital, 35-1 Chigasaki-chuo, Tsuzuki-ku, Yokohama, Kanagawa pref. 224-8503, Japan

**Keywords:** Malignant peritoneal mesothelioma, localized mesothelioma, transverse colon

## Abstract

**Background:**

We report an extremely rare case of resection of localized biphasic malignant peritoneal mesothelioma of the transverse colon.

**Case report:**

Computed tomography and magnetic resonance imaging in a 72-year-old man showed a tumor with enhanced borders consistent with the transverse colon. Colonoscopy showed ulcerative lesions in the transverse colon, but histological examination showed no malignancy. A gastrointestinal stromal tumor was strongly suspected, so an extended right hemicolectomy was performed. Histopathological examination showed that the tumor was a localized malignant peritoneal mesothelioma of the transverse colon. The patient did not receive postoperative chemotherapy and died 18 months after surgery.

**Conclusions:**

The number of patients with malignant mesotheliomas is predicted to increase in the future both in Japan and in western countries. We report this case due to its probable usefulness in future studies pertaining to the diagnosis and treatment of malignant mesotheliomas.

## Background

Malignant mesotheliomas are rare tumors that reportedly account for 0.2% of all malignant tumors [1,2]. Malignant peritoneal mesotheliomas occurring in the peritoneum have an even lower incidence. Most reported cases of malignant peritoneal mesothelioma are diffuse type, and localized cases are rare [3,4]. In this report, we describe our experience with a case of resection of a localized malignant peritoneal mesothelioma that had developed within the visceral fascia of the transverse colon.

## Case presentation

Our patient was a 72-year-old man who had been diagnosed as a rectal cancer 10 years prior to admission, and was treated surgically by low anterior resection at that time. His occupational and residence history showed no apparent exposure to asbestos. Four months before admission, the patient noticed a mass in his upper abdomen; two months before admission, he experienced abdominal pain.

**Figure 1:**
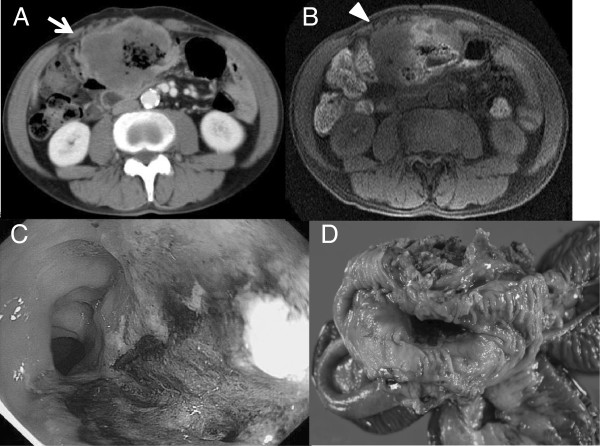
**Tumor mass surrounded the transverse colon in the epigastric region. A**. Enhanced computed tomography (CT) showed internal content was homogenous, and only its margins were contrast-enhanced (arrow). **B**. Enhanced magnetic resonance imaging (MRI) showed only the margins were enhanced (arrow head). **C**. Colonoscopy revealed an ulcerated lesion in the transverse colon and the ulcer base was dark brown due to the adherence of iron from the patient’s medication. **D**. The resected specimen showed a tumor which had initially developed in the transverse colon, had infiltrated the ileum.

On admission, a fist-sized tumor in the epigastric region, as well as abdominal tenderness, was noted. Laboratory findings showed a hemoglobin level of 8.6 g/dL and a C-reactive protein level of 5.5 mg/dL, which were indicative of anemia and inflammation, respectively. Tumor marker levels were all within the reference values: carcinoembryonic antigen (CEA), 0.9 ng/mL (<5.0 ng/mL); cancer antigen 19-9, 2.6 U/mL (<37.0 U/mL); α-fetoprotein, 1.5 ng/mL (<10.0 ng/mL); sialyl Tn antigen, 17.6 U/mL (<45.0 U/mL); and cancer antigen 72-4, 2.1 U/mL (<6.9 U/mL). Computed tomography (CT) scans showed no abnormal findings in the mediastinum or lung fields, as well as no pleural hypertrophy or nodules. A tumor mass surrounding the transverse colon was found in the epigastric region; its internal content was homogenous, and only its margins were contrast-enhanced (Figure 1A). The lumen of the transverse colon was preserved. There was no pleural effusion or ascites, and no nodular lesions in the chest or abdomen were observed. Similar to the CT findings, magnetic resonance imaging (MRI) showed a tumor in the epigastric region in which only the margins were contrast-enhanced (Figure 1B). Colonoscopy revealed an ulcerated lesion in the transverse colon with a 5-cm major axis and without a rand wall, which occupied two-thirds of the diameter of the intestinal lumen. The central portion of the lesion formed a deep ulceration and the ulcer base was dark brown, which was believed to be due to the adherence of iron from the patient’s medication (Figure 1C). A biopsy showed only a hyperplastic mucosa, necrotic tissue, and extensive infiltration of inflammatory cells; there was no apparent neoplastic lesion. Fluorodeoxyglucose-positron emission tomography (PET) tests showed a strong abnormal uptake of the contrast material by the tumor in the epigastric region, which was detected by CT and MRI. No abnormal uptake was found in any other location.

**Figure 2:**
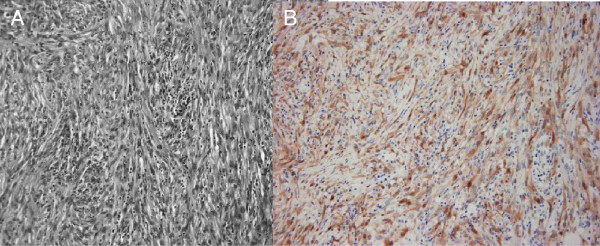
**Histopathologically, the tumor was diagnosed localized biphasic malignant peritoneal mesothelioma. A**. Tumor was composed of spindle cells with a mitoses as well as epithelioid cells showing fasciculated growth (hematoxylin and eosin (H. E.) staining × 200). **B**. Immunohistochemical staining showed that the calretinin were positive (×200).

A gastrointestinal stromal tumor was suspected, and therefore, a laparotomy was performed. The surgical findings showed a tumor in the transverse colon as well as extensive infiltration of the ileum. An extended right hemicolectomy and partial ileal resection of the infiltrated regions were performed. No ascites or disseminated tumors were found in the abdominal cavity. The resected specimen showed a tumor (dimensions: 10 × 9 × 5 cm) that formed an ulcer on the mucosa of the transverse colon. Macroscopic examination showed that the tumor, which had initially developed in the transverse colon, had infiltrated the ileum (Figure 1D). Histopathologically, hematoxylin-eosin staining showed that the tumor was composed of spindle cells with a mitoses as well as epithelioid cells showing fasciculated growth (Figure 2A). Immunohistochemical staining showed that the calretinin (+), AE1/AE3 (cytokeratin) (+), vimentin (++), HBME-1 (+), α-SMA (++), desmin (-), S100 (-), c-kit (-), CD34 (-), and Ki-67 antibody labeling index was high (Figure 2B). Comprehensively, the diagnosis was localized biphasic malignant peritoneal mesothelioma that had initially developed in the visceral fascia of the transverse colon. Similar histological aspects were found in 6 of the 16 dissected lymph nodes that were diagnosed as metastasis. The patient did not receive postoperative chemotherapy, and the follow-up was conducted in an outpatient setting. Recurrence of the peritoneal metastasis was found 7 months after the surgery; the recurrent tumor increased in size and the patient died 18 months after the surgery.

## Discussion

Malignant mesotheliomas account for approximately 0.2% of all malignant tumors, and malignant peritoneal mesotheliomas are even more rare [1,2]. We conducted a search on Pub-Med using the keyword ‘malignant peritoneal mesothelioma’, and the organs that were mentioned as the primary tumor sites in the search results included the colon in five cases, spleen in one, stomach in one, liver in two, and prostate in one. These findings suggest that this disease is extremely rare.

Among the primary sites of malignant mesothelioma, the pleural membranes account for 80 to 90% of cases; cases of malignant mesothelioma developing from the peritoneum are as rare (apprroximately5 to 10%) [3,4]. In addition, they are macroscopically classified as diffuse and localized malignant mesotheliomas. Diffuse malignant mesotheliomas account for 85% of such cases, and resectable localized cases are very rare [5]. Histologically, they are classified as the epithelioid, biphasic, or sarcomatoid type. The epithelioid type accounts for approximately 75% of malignant mesotheliomas, whereas the sarcomatoid type is the rarest type of malignant mesothelioma and has a poor prognosis [3,4].

Exposure to asbestos has been found to be a factor contributing to the development of mesotheliomas. However, patients with a well-defined history of exposure account for approximately 90% of cases of pleural mesotheliomas but only for 20 to 50% of cases of peritoneal mesotheliomas [6]. In addition, chronic serositis, viral infections (simian virus 40), and a history of radiation therapy are also presumed to be involved in the development of the disease, but these hypotheses lack scientific evidence [7].

This disease has no specific subjective symptoms. In some cases, laboratory findings may also include increased levels of CYFRA and hyaluronic acid in pleural effusions and ascites fluid, CEA levels within the normal range, and increased levels of inflammatory proteins, as well as thrombocytosis due to the production of interleukin 6 from peritoneal mesotheliomas [8]. In some cases, diagnostic imaging such as CT, MRI, and PET may provide useful information for reference, but in most cases, the definitive diagnosis is obtained by tumor resection, as in the case described in our report [3,4].

Surgery is the first choice treatment for localized tumors; however, recurrence occurs a few months after surgery and long-term survival is rarely achieved [5]. Particularly for localized tumors, patients with positive lymph node metastasis are believed to have a poor prognosis despite tumor resection [9]. In addition, most malignant peritoneal mesotheliomas are diffuse and difficult to resect, and therefore, in most cases, the treatment consists mainly of chemotherapy [10]. In terms of chemotherapy, multiple drug regimens consisting mainly of cisplatin (CDDP) are widely used. However, various regimens such as CDDP + CPT-11 and CDDP + Mitomycin C or CDDP + pemetrexed have been used, but the response rate is approximately 25 to 40% [7,11]. In some cases, prolonged survival has been achieved using these treatments, but as a whole, the mean survival period is approximately 12 months and the prognosis is very poor [9].

The causes, living history, and environmental conditions that are likely to be associated with asbestos exposure have recently been elucidated [4]. Given that the onset of malignant mesothelioma occurs 30 to 40 years after asbestos exposure and according to the past use of asbestos, it is estimated that the peak of the frequency of malignant mesothelioma will be reached in the 2020s in Europe and in the 2010s in Australia [12,13], whereas the peak is believed to have passed already in the USA, where usage regulations were changed earlier [14]. In Japan, the peak is predicted to be reached in approximately 2030 [15].

## Conclusions

The number of patients with malignant mesothelioma is estimated to increase in the future; therefore, there will be an increasing number of opportunities to experience cases of localized malignant peritoneal mesothelioma, such as the our case. Studies containing larger numbers of cases are needed to improve the prognosis. We believe that this report will be useful for the future clinical diagnosis and treatment of the disease.

## Consent

Written informed consent was obtained from the patient’s family for the publication of this case report and any accompanying images. A copy of the written consent is available for review by the Editor-in-Chief of this journal.

## Abbreviations

CEA: carcinoembryonic antigen; CT: computed tomography; H.E.: hematoxylin and eosin; MRI: magnetic resonance imaging; PET: positron emission tomography.

## Competing interests

The authors declare that they have no competing interests for this report.

## Authors’ contributions

YT was responsible for the writing. SE, KN and DT participated in data collection. YM performed endoscopy and reviewed the images SS, TO, CM, SM, EH, FI, JT and SK participated in literature searching. All authors read and approved the final manuscript.
